# COVID-19-related acute kidney injury; incidence, risk factors and outcomes in a large UK cohort

**DOI:** 10.1186/s12882-021-02557-x

**Published:** 2021-11-01

**Authors:** Paul D. Jewell, Kate Bramham, James Galloway, Frank Post, Sam Norton, James Teo, Richard Fisher, Rohit Saha, Sam Hutchings, Phil Hopkins, Priscilla Smith, Jennifer Joslin, Satish Jayawardene, Sarah Mackie, Ali Mudhaffer, Amelia Holloway, Henry Kibble, Mosammat Akter, Benjamin Zuckerman, Kieran Palmer, Ciara Murphy, Domniki Iatropoulou, Claire C. Sharpe, Eirini Lioudaki

**Affiliations:** 1 Renal Unit, King’s College Hospital NHS Foundation Trust, Denmark Hill, London, SE5 9RS UK; 2 Faculty of Life Sciences and Medicine, King’s College London, London, UK; 3 Centre for Rheumatic Disease, King’s College London, London, UK; 4 Department of Sexual Health and HIV, King’s College Hospital NHS Foundation Trust, London, UK; 5 Department of Neurosciences, King’s College Hospital NHS Foundation Trust, London, UK; 6 Department of Critical Care, King’s College Hospital NHS Foundation Trust, London, UK

**Keywords:** COVID-19, AKI, Mortality, Renal replacement therapy

## Abstract

**Background:**

Acute kidney injury (AKI) is common among patients hospitalised with COVID-19 and associated with worse prognosis. The aim of this study was to investigate the epidemiology, risk factors and outcomes of AKI in patients with COVID-19 in a large UK tertiary centre.

**Methods:**

We analysed data of consecutive adults admitted with a laboratory-confirmed diagnosis of COVID-19 across two sites of a hospital in London, UK, from 1st January to 13th May 2020.

**Results:**

Of the 1248 inpatients included, 487 (39%) experienced AKI (51% stage 1, 13% stage 2, and 36% stage 3). The weekly AKI incidence rate gradually increased to peak at week 5 (3.12 cases/100 patient-days), before reducing to its nadir (0.83 cases/100 patient-days) at the end the study period (week 10). Among AKI survivors, 84.0% had recovered renal function to pre-admission levels before discharge and none required on-going renal replacement therapy (RRT). Pre-existing renal impairment [odds ratio (OR) 3.05, 95%CI 2.24–4,18; *p* <  0.0001], and inpatient diuretic use (OR 1.79, 95%CI 1.27–2.53; *p* <  0.005) were independently associated with a higher risk for AKI. AKI was a strong predictor of 30-day mortality with an increasing risk across AKI stages [adjusted hazard ratio (HR) 1.59 (95%CI 1.19–2.13) for stage 1; *p* < 0.005, 2.71(95%CI 1.82–4.05); *p* < 0.001for stage 2 and 2.99 (95%CI 2.17–4.11); p < 0.001for stage 3]. One third of AKI3 survivors (30.7%), had newly established renal impairment at 3 to 6 months.

**Conclusions:**

This large UK cohort demonstrated a high AKI incidence and was associated with increased mortality even at stage 1. Inpatient diuretic use was linked to a higher AKI risk. One third of survivors with AKI3 exhibited newly established renal impairment already at 3–6 months.

**Supplementary Information:**

The online version contains supplementary material available at 10.1186/s12882-021-02557-x.

## Introduction

Since its emergence in December 2019, Coronavirus disease-2019 (COVID-19) caused by the newly recognised severe acute respiratory syndrome coronavirus-2019 (SARS-CoV-2), has affected millions of individuals worldwide, with considerable morbidity and mortality. Despite initial reports of low incidence [1–4], acute kidney injury (AKI) in patients hospitalised with COVID-19 has now been recognised as an important disease complication. So far, evidence is predominantly sourced from large US series [5, 6] while UK data are limited [7]. The presence of AKI is associated with higher mortality risk in the context of acute respiratory distress syndrome (ARDS) of all aetiologies [8] and, although causality is not established, AKI has been linked to an increased risk of chronic kidney disease (CKD) in individuals with previously normal renal function [9].

In light of potential short- and long-term implications, it is important to understand the epidemiology of all AKI stages in patients with COVID-19 globally but also locally and identify potential risk factors. These findings may provide meaningful insight, with regards to resource allocation, clinical management and research planning during the next phases of the pandemic. Within our centre, one of London’s busiest covering a 1.2 million population area, we care for a diverse population, and we continue to manage a substantial number of patients with COVID-19.

The aim of this study was to describe the incidence of different stages of AKI in patients admitted to our centre with COVID-19, identify risk factors and explore mortality and renal specific outcomes in patients with COVID-19 related AKI.

## Methods

### Study design

We performed a retrospective cohort study across two sites of King’s College Hospital, London, to investigate AKI in patients admitted with COVID-19. The hospital serves a multi-ethnic population of approximately 1.2 million. The study was performed in accordance with the principles of the Declaration of Helsinki and under London South East Research Ethics Committee (reference 18/LO/2048) approval granted to the King’s Electronic Records Research Interface (KERRI); specific work on COVID19 research was reviewed in March 2020 and reaffirmed in April 2020 with expert patient input on a virtual committee with Caldicott Guardian oversight. Additional lawful data processing was permitted under, the Health Service Control of Patient Information Regulations 2002 (COPI) Notice, which was issued by the UK Secretary of State for Health and Social Care in March 2020 providing a legal basis for processing confidential patient information without consent for Covid-19 healthcare.

### Data acquisition and study population

All adult hospital admissions with a positive nasopharygeal swab test for SARS-CoV-2 from 01 January to 13 May 2020 were screened for suitability (not including inter-hospital transfers due to lack of information). Patients who required admission with a clinical presentation consistent with COVID-19, and a first positive SARS-CoV-2 swab test on, or at up to 7 days after admission were suitable for inclusion in the analysis. Patients with probable hospital-acquired COVID-19 as defined by a first positive swab test occurring on or after the 8th day of hospitalisation [10] or end-stage renal disease (ESRD) already requiring renal replacement therapy (RRT) including all patients with kidney transplants, were excluded. Kidney transplant patients were excluded because of different physiology, patient characteristics and susceptibility to kidney insult as compared to non-transplant AKI. Patients who were transferred between the two hospital sites were treated as one admission. When more than one admission for one patient was identified, the first hospitalisation (chronologically related to first positive swab) was included.

Data were obtained from the Electronic Health Records (EHR; Sunrise Clinical Manager-Allscripts, Renalware and IntelliSpace Critical Care and Anaesthesia, Philipps). Demographics included age, gender and self-identified ethnicity coded as White, Black, Asian, or other (including “mixed”). We collected data on comorbidities (hypertension, diabetes, cardiovascular disease including ischaemic heart disease and heart failure, malignancy, neurological disease including dementia, lung disease and smoking status),admission laboratory results (haemoglobin, neutrophil:lymphocyte ratio, platelet count, urea, sodium, albumin, CRP) and chest radiograph, peak results of D-dimer, troponin and creatine kinase (CK) during admission, medication history (specifically ACE inhibitor (ACEi) or angiotensin-receptor blocker (ARB) usage and immunosuppression), inpatient diuretic administration (use of oral or intravenous diuretics for at least 24 h), inpatient diagnosis of deep vein thrombosis (DVT) or pulmonary embolism (PE), and admission to intensive care unit (ICU). Admission chest radiograph is defined as abnormal if the radiologist’s report is consistent with features of COVID-19. Serial creatinine measurements were recorded for up to the first 30 days of hospitalisation and on discharge. A history of CKD was validated by historical estimated glomerular filtration rate (eGFR) (see below). Information on historical creatinine measurements were collected individually from EHR. Outcome data were recorded, including need for RRT and inpatient mortality and/or at 30 days. The RRT modalities used included continuous veno-venous haemodiafiltration (CVVHDF), intermittent haemodialysis (HD) and peritoneal dialysis (PD). Additional data for patients admitted to ICU were also collected [use of mechanical ventilation, extracorporeal membrane oxygenation (ECMO), and vasopressor support].

### Definitions and calculations

Diagnosis and stage of CKD were defined according to Kidney Disease Improving Global Outcomes (KDIGO) criteria [11]. The diagnosis of AKI was made using KDIGO criteria [12] and classified accordingly as stage 1 (increase in serum creatinine of ≥26.5micromol/l or 1.5–1.9 times baseline creatinine), stage 2 (increase to 2.0–2.9 times from baseline) or stage 3 (increase > 3 times baseline serum creatinine or a peak serum creatinine ≥353.6 μmol/l or initiation of RRT). Urine output was not used as part of the definition as it is not recorded in EHR. Historical baseline creatinine was recorded as the most recent measurement within 8 to 365 days prior to hospital admission. When this was not available and there was no documentation of CKD, the lowest creatinine during admission in the absence of RRT was recorded as baseline provided this was within the normal range. If the nadir creatinine was not within the normal range and there was no record of CKD, baseline creatinine was imputed based on gender and race using the Modification of Diet in Renal Disease (MDRD) Study equation assuming that baseline eGFR is 75 ml/min per 1.73m^2^ [11]. Baseline eGFR was calculated using the MDRD formula and the recorded baseline creatinine value. Weekly incidence rates of AKI were calculated using new cases of AKI during each seven-day time period as the numerator, and the number of patients susceptible to AKI during the seven-day time period as the denominator. These rates were then converted and presented as rates per 100-person days.

Recovery from AKI was defined as the absence of any stage AKI in the last recorded creatinine during hospitalisation (i.e. serum creatinine < 1.5 times the baseline creatinine), in the absence of RRT [13, 14]Follow-up creatinine data at 3 to 6 months were collected for patients with an AKI stage 3.

### Outcomes

The outcomes were the development of AKI, RRT requirements, AKI recovery and mortality.

### Statistical methods

Numerical data are expressed as mean with standard deviation (SD) or median with interquartile range (IQR) where appropriate. Categorical data are presented as absolute number with percentage. Comparison between groups was performed using Chi-squared or Fisher exact test for categorical variables, and Student’s t-test or Wilcoxon rank test for continuous variables. All tests were two-sided and a *p*-value< 0.05 was considered as significant. Binary logistic regression was used to identify factors associated with AKI with odds ratios (ORs) and 95% confidence intervals presented (CIs). Cox proportional hazards regression was used to perform a time-to-event analysis for 30-day mortality (defined as death within 30 days from date of admission). Hazard ratios (HRs) and 95% CIs are presented and supplemented by Kaplan-Meier survival curves to demonstrate the effect of AKI on the outcome. For both the logistic and Cox regression analyses, initially a single variable analysis was used to determine factors associated with outcome unadjusted for other variables. Where the association was significant at *p* < 0.10, the variables were included in multivariable models to determine association with the outcome adjusting for potential confounders. This included age, ethnicity, comorbidities (CKD, hypertension, diabetes, neurological and lung disease) and laboratory results (albumin, CRP, neutrophil:lymphocyte ratio). Missing data were excluded from the analyses: 52 (4.1%) and 39 (3.1%) observations in the logistic and Cox multivariable regression models respectively. Imputation of missing data was not performed due to small volume of missing data. R statistical package version 1.4.1106 was used for all statistical analysis.

## Results

### AKI incidence and patient characteristics

**Fig. 1:**
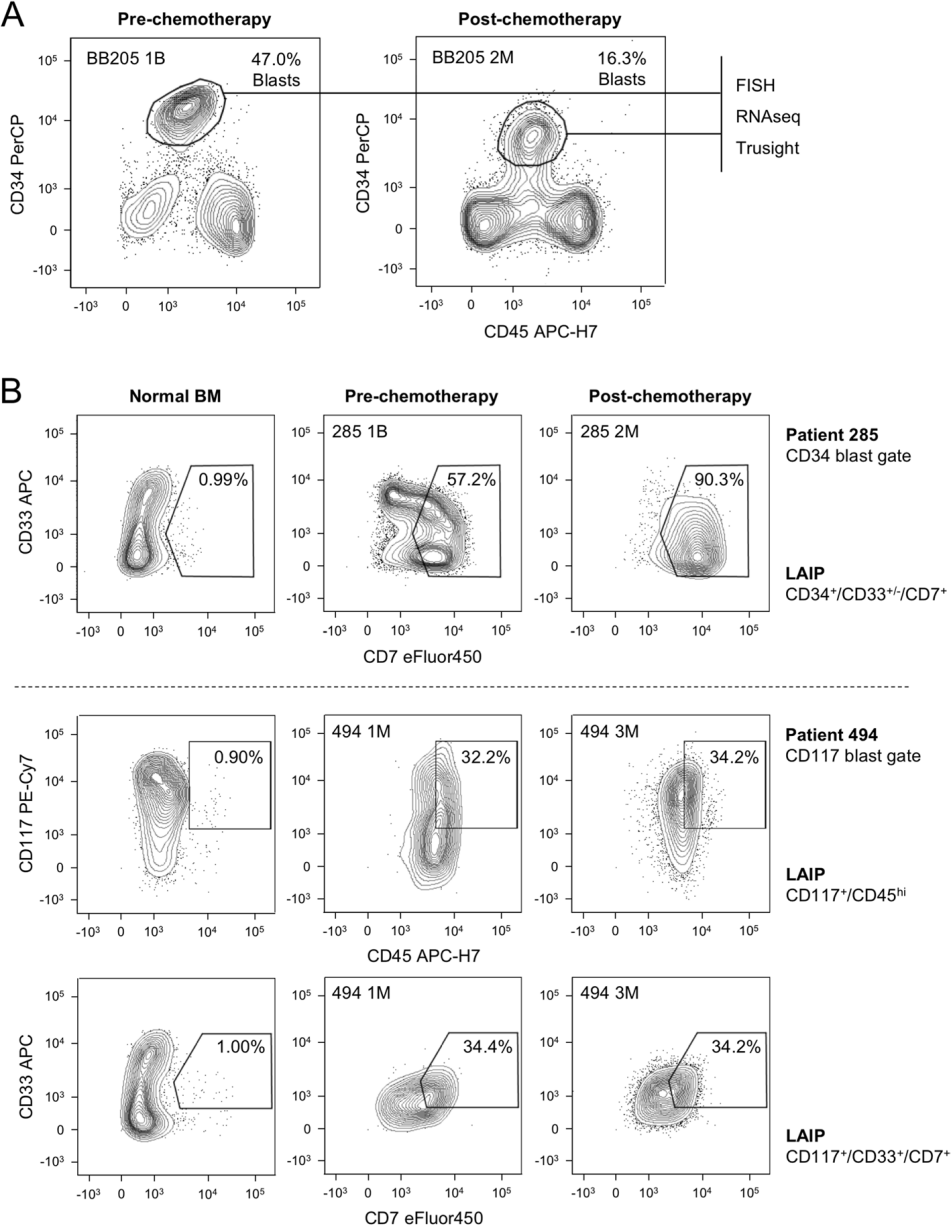
Weekly acute kidney injury (AKI) incidence rate

A total of 1248 patients were included in the final analysis (Suppl. material Fig. 1). Demographics and baseline clinical characteristics are described in Table 1. Mean age was 69 years (SD 17.1), 58.8% were male, 49.1% were of white and 27.4% of black ethnicity. A diagnosis of pre-existing CKD stages 3–5 (baseline eGFR< 60 ml/min/1.73m^2^) was present in 16.6%; 14.0% had CKD stage 3, 2.1% CKD stage 4 and 0.5% CKD stage 5 (not on RRT).

**Table 1:** Baseline and admission characteristics

	All (***n*** = 1248)	No AKI (***n*** = 761)	All AKI (***n*** = 487)	AKI1 (n = 248)	AKI2 (n = 64)	AKI3 (n = 175)	p^b^	p^c^ for trend
**Demographics**								
**Age** (years) mean (SD)	69 (17.1)	67.4 (18.2)	71.2 (14.7)	74.4 (14.4)	76.3 (13.2)	64.7 (13.4)	0.002	< 0.001
**Male,** n (%)	734 (58.8%)	423 (55.6%)	311 (63.9%)	150 (60.5%)	40 (62.5%)	121 (69.1%)	0.004	0.009
**Ethnicity,** n (%)							0.003	< 0.001
White	613 (49.1%)	392 (51.5%)	221 (45.4%)	130 (52.4%)	36 (56.2%)	55 (31.4%)		
Black	342 (27.4%)	183 (24%)	159 (32.6%)	69 (27.8%)	20 (31.2%)	70 (40.0%)		
Asian	102 (8.2%)	69 (9.1%)	33 (6.8%)	14 (5.6%)	4 (6.2%)	15 (8.6%)		
Mixed and other	81 (6.5%)	52 (6.8%)	29 (5.9%)	10 (4.0%)	2 (3.1%)	17 (9.8%)		
Unknown	110 (8.8%)	65 (8.5%)	45 (9.2%)	25 (10.1%)	2 (3.1%)	18 (10.3%)		
**Comorbidities**								
**CKD**^**a**^**,** n (%)	207 (26.6%)	77 (10.1%)	130 (26.7%)	75(30.2%)	18(28.2%)	37(21.1%)	< 0.001	< 0.001
**Hypertension,** n (%)	681 (54.6%)	346 (45.5%)	335 (68.8%)	176 (71.0%)	44 (68.8%)	115 (65.7%)	< 0.001	< 0.001
**IHD,** n (%)	180 (14.5%)	101 (13.4%)	79 (16.2%)	43 (17.3%)	11 (17.2%)	25 (14.3%)	0.162	0.426
**Heart failure,** n (%)	161 (12.9%)	79 (10.4%)	82 (16.8%)	50 (20.2%)	13 (20.3%)	19 (10.9%)	< 0.001	< 0.001
**Diabetes,** n (%)	406 (32.7%)	207 (27.4%)	199 (40.9%)	100 (40.3%)	25 (39.7%)	74 (42.3%)	< 0.001	< 0.001
**Malignancy,** n (%)	207 (16.6%)	122 (16.0%)	85 (17.5%)	53 (21.4%)	13 (20.6%)	19 (10.9%)	0.500	0.027
**Neurological history,** n (%)							0.071	< 0.001
Dementia	203 (16.3%)	112 (14.7%)	91 (18.7%)	61 (24.6%)	16 (25.0%)	14 (8.0%)		
Other	163 (13.1%)	109 (14.3%)	54 (11.1%)	30 (12.1%)	9 (14.1%)	15 (8.6%)		
**Lung disease,** n (%)							0.007	0.028
Asthma	118 (9.5%)	85 (11.2%)	33 (6.8%)	16 (6.5%)	2 (3.1%)	15 (8.6%)		
COPD	122 (9.8%)	70 (9.2%)	52 (10.7%)	28 (11.3%)	9 (14.1%)	15 (8.6%)		
Other	75 (6%)	36 (4.7%)	39 (8.0%)	23 (9.3%)	2 (3.1%)	14 (8.0%)		
**Smoking status,** n (%), (*n* = 296)							0.101	0.252
Current	41(4.3%)	23 (3.9%)	18 (4.9%)	12 (6.2%)	2 (4.4%)	4 (3.1%)		
Ex	287 (30.1%)	164 (27.9%)	123 (33.7%)	68 (35.4%)	16 (35.6%)	39 (30.5%)		
**Medications**								
**ACEi or ARB usage**, n (%)	331 (26.5%)	161 (21.2%)	170 (34.9%)	92 (37.1%)	20 (31.2%)	58 (33.1%)	< 0.001	< 0.001
**Immunosuppression,** n (%)	120 (9.7%)	71 (9.4%)	49 (10.1%)	25 (10.1%)	9 (14.1%)	15 (8.6%)	0.679	0.624
**Laboratory results**								
**Neutrophil:Lymphoyte ratio**, Median (IQR)	5.6 (3.6, 9.5)	5.2 (3.3, 8.6)	6.5 (4.1, 10.9)	6.1 (3.8, 10.6)	6.0 (3.5, 14.0)	7.4 (4.7, 11.1)	< 0.001	< 0.001
**CRP** (mg/l), Median (IQR)	84 (38.9, 151)	74 (33.4, 133)	107.8 (49.4, 170.3)	80 (41.3, 142.0)	108.2 (40.4, 169.6)	136.0 (79.7, 241.4)	< 0.001	< 0.001
**Albumin**(g/l), Median (IQR)	37 (34.0, 40)	38 (35.0, 41)	37 (34.0, 39.0)	37 (34, 39.5)	36 (31.5, 39)	37 (34.0, 39)	< 0.001	< 0.001
**Haemoglobin**(g/dl), Median (IQR)	133 (120, 145)	133 (121, 144)	132 (116, 145)	127 (112, 141)	131 (115, 145)	138.0 (124, 152)	0.125	< 0.001
**Platelet count**(×10^9^ /l), Median (IQR)	216(165, 274)	214(168, 270)	217 (162, 279)	223 (162, 283)	215 (161, 275)	212 (163, 278)	0.882	0.926
**Clinical characteristics**								
**Inpatient diuretic use,**n (%)	199 (16.1%)	91 (12.1%)	108 (22.3%)	64 (25.8%)	14 (21.9%)	30 (17.4%)	< 0.001	< 0.001
**Inpatient PE or DVT,** n (%)	86 (6.9%)	33 (4.3%)	53 (10.9%)	13 (5.3%)	5 (7.8%)	35 (20.1%)	< 0.001	< 0.001
**ICU admission,** n (%)	192 (15.4%)	43 (5.7%)	149 (30.6%)	16 (6.5%)	7 (10.9%)	126 (72%)	< 0.001	< 0.001

*AKI* acute kidney injury, *CKD* chronickidneydisease, *IHD* ischaemic heart disease, *COPD* chronic obstructive pulmonary disease, *ACE-I* angiotensin-converting enzyme inhibitor, *ARB* angiotensin receptor blocker, *PE* pulmonary embolism, *DVT* deep vein thrombosis, *ICU* intensive care unit

^a^defined as eGFR< 60 ml/min/1.73m^2^

^b^comparison between AKI vs non-AKI

^c^comparison across AKI stage subgroups

A total of 487 patients (39%) developed AKI and the proportion of these with pre-existing CKD was significantly higher at 26.7%, compared to those without AKI (10.1%, *p* < 0.001). Hypertension (54.6%) and diabetes (32.7%) were the most common comorbidities and were both significantly more frequent among patients with AKI (68.8 and 40.9%, respectively) than those without (45.5 and 27.4%, respectively, *p* < 0.001for both) (Table 1).

Admission laboratory results, clinical characteristics and outcomes are described in Table 1 and Suppl.Table 1. Inpatient diuretics were more frequently used among patients with AKI compared to those without (22.3 vs 12.1%; *p* < 0.001). Patients who developed AKI more commonly suffered an acute venous thromboembolism (10.9% vs 4.3%, p < 0.001), were admitted to ICU (30.6% vs 5.7% without AKI, p < 0.001) and/or died compared to those who did not (42.1% vs 16.8%, p < 0.001).

The weekly incidence rate increased over time to peak at week 5 i.e., midpoint of observation period (3.12 new cases per 100 patient-days) and then gradually declined to reach a nadir at week 10 (end of observation period) (0.83 new cases per 100 patient-days) (Fig. 1).

### Renal function and outcomes

Renal outcomes are presented in Table 2. Of the 487 cases of AKI, 51% met KDIGO criteria for stage 1 (*n* = 248), 13% (*n* = 64) stage 2 and 36% (*n* = 175) stage 3. A total of 109 patients (8.7% of total, 22% of all AKI) required RRT and the relevant modalities included CVVHDF, HD, PD or any combination of those (different modalities for a single patient were used at different times and according to clinical indication and resource availability) [15]. None of the patients required RRT following discharge from the hospital. Of those discharged alive, AKI had resolved in 84.0% of all patients affected, and 69.7% of those with AKI3 prior to discharge.

**Table 2:** Renal characteristics and outcomes

	All (n = 1248)	No AKI (n = 761)	All AKI (n = 487)	AKI 1 (n = 248)	AKI 2 (n = 64)	AKI 3 (n = 175)	p^b^	p^c^ for trend
**Baseline Cr** (μmol/l)**,** Median (IQR)	81.0 (66.0, 99.0)	77.0 (64.0, 92.0)	91.0 (72.0, 116.5)	95.0 (75.0, 121.8)	85.0 (70.0, 118.2)	85.0 (70.0, 106.0)	< 0.001	< 0.001
**Baseline eGFR** **(**ml/min/1.73m^2^)**,**Median (IQR)	79.0 (61.4, 97.7)	82.7 (68.5, 100.7)	69.8 (48.2, 90.8)	64.4 (44.9, 85.9)	63.4 (50.2, 101.8)	77.5 (59.7, 96.8)	< 0.001	< 0.001
**Admission** (μmol/l), Median (IQR)	91.0 (71.0, 126.5)	80.0 (65.0, 96.0)	132 (98.0, 207.)	129 (101.0, 176.0)	184(117.2, 261.5)	121(87.5, 242.5)	< 0.001	< 0.001
**Peak Cr** (μmol/l)**,** Median (IQR)	98.0 (74.8, 162.0)	81.0 (66.0, 97.0)	199.0 (134.0, 367.5)	140 (114.8, 185.8)	203.5 (165.5, 275.5)	462.(285.5, 631.0)	< 0.001	< 0.001
**Urine protein**^**a**^ (*n* = 287)	123 (42.9%)	53 (35.3%)	70 (51.1%)	26 (38.2%)	13 (68.4%)	31 (62.0%)	0.007	< 0.001
**Urine RBCs**^**a**^ (*n* = 331)	129 (39.0%)	58 (33.0%)	71 (45.8%)	31 (39.2%)	14 (66.7%)	26 (47.3%)	0.017	0.011
**RRT,**n (%)	109 (8.7%)	n/a	109 (22.4%)	0 (0.0%)	0 (0.0%)	109 (62.3%)	< 0.001	< 0.001
**RRT modality,**n (%)								
CVVHDF	63 (5.0%)	n/a	63 (12.9%)	n/a	n/a	63 (36.0%)		
CVVHDF/HD	12 (1.0%)	n/a	12 (2.5%)	n/a	n/a	12 (6.9%)		
CVVHDF/PD	26 (2.1%)	n/a	26 (5.3%)	n/a	n/a	26 (14.9%)		
CVVHDF/PD/HD	4 (0.3%)	n/a	4 (0.8%)	n/a	n/a	4 (2.3%)		
HD	4 (0.3%)	n/a	4 (0.8%)	n/a	n/a	4 (2.3%)		
None	1139 (91.3%)	n/a	378 (77.6%)	n/a	n/a	66 (37.7%)		
**RRT off ICU,** n (%)	14 (1.1%)	n/a	14 (2.9%)	n/a	n/a	14 (8.0%)		
**RRT after discharge,** n (%)	0 (0.0%)	n/a	0 (0.0%)	n/a	n/a	0 (0.0%)		
**Discharge Cr** (μmol/l)**,** Mean (SD)	80.0 (63.0, 114.0)	70.0 (58.0, 85.0)	124.0 (81.5, 212.5)	105.0 (79.0, 160.0)	148.0 (73.0, 202.0)	186.0 (96.0, 359.0)	< 0.001	< 0.001
**Cr recovered to baseline,** n (%) (alive patients only *n* = 915)	n/a	n/a	219 (84.6%)	143 (89.9%)	24 (85.7%)	52 (72.2%)		< 0.001
**Admission outcome,** n (%)								
Death in hospital	348 (27.9%)	132 (17.3%)	216 (44.4%)	89 (35.9%)	35 (54.7%)	92 (52.6%)	< 0.001	< 0.001
Discharged alive	900 (72.1%)	629 (82.7%)	271 (55.6%)	159 (64.1%)	29 (45.3%)	83 (47.4%)	< 0.001	< 0.001

*AKI* acute kidney injury, *Cr* creatinine, *IQR* interquartile range, *eGFR* estimated glomerular filtration rate, *ICU* intensive care unit, *RBCs* red blood cells, *RRT* renal replacement therapy; *CVVHDF* continuousveno-venous haemodiafiltration, *HD* haemodialysis, *PD* peritoneal dialysis, *SD* standard deviation

^a^in urine dip

^b^comparison between AKI vs non-AKI

^c^comparison across AKI stage subgroups

Follow up data at 3 to 6 months were available for 78.3% of survivors with AKI stage 3 (65 out of 83), the vast majority of which (80%) had no pre-existing renal impairment. An eGFR drop from baseline equal or higher than 15 ml/min/1.73m^2^ was noted in 30.7%, while 21.5% presented an eGFR newly established at below 60 ml/min/1.73m^2^.

### Binary logistic regression for AKI

Multivariate logistic regression analysis revealed that pre-existing CKD (baseline eGFR< 60 ml/min/1.73m^2^) was associated with a 3-fold risk of AKI (OR 3.05; 95%CI 2.24–4.18, *p* < 0.0001) adjusted for demographics and comorbidities (Table 3). Other variables independently associated with increased AKI risk were male sex (OR 1.45; 95%CI 1.12–1.89, *p* = 0.005), black ethnicity (OR 1.76; 95%CI1.26–2.45, *p* < 0.005), hypertension (OR 1.66; 95%CI1.23–2.24, p < 0.005) and inpatient diuretic use (OR 1.79; 95%CI 1.27–2.53, p < 0.005).

**Table 3:** Univariate and multivariate logistic regression analyses of risk factors associated with the development of AKI

Variable	Unadjusted OR	95% CI	***p*** value	Adjusted^**a**^OR	95% CI	p value
**Age**	1.01	1.01–1.02	0.0002	1.00	1.03–1.05	0.4483
**Male sex**	1.41	1.11–1.79	0.0038	1.45	1.12–1.8	0.0055
**Race: White ethnicity**	Ref	Ref	Ref	Ref	Ref	Ref
**Black**	1.54	1.18–2.02	0.0016	1.76	0.66–1.20	0.0009
**Asian**	0.85	0.54–1.32	0.4701	1.05	0.57–1.38	0.8584
**Mixed/Other**	0.99	0.60–1.59	0.9649	1.21	0.6701.85	0.4904
**Unknown**	1.23	0.81–1.85	0.3313	1.56	0.52–1.35	0.0615
**CKD**	3.52	2.69–4.63	< 0.001	3.05	1.11–1.82	0.0000
**Hypertension**	2.64	2.08–3.35	< 0.001	1.66	0.68–1.11	0.0010
**Diabetes**	1.84	1.44–2.34	< 0.001	1.20	0.78–1.26	0.2028
**CVD**	1.53	1.20–1.95	0.0006	1.10	0.82–1.48	0.5145
**Lung disease**	1.02	0.78–1.32	0.8958	n/a	n/a	
**Malignancy**	1.11	0.82–1.50	0.4998	n/a	n/a	
**ACEi/ARB use**	2.00	1.55–2.58	< 0.001	1.26	0.93–1.71	0.1309
**Inpatient diuretic use**	2.09	1.54–2.84	< 0.001	1.79	1.27–2.53	0.0009
**Neutrophil: Lymphocyte ratio**	1.03	1.02–1.05	< 0.001	1.00	0.99–1.02	0.0332
**Haemoglobin**	1.00	0.99–1.0	0,1657	n/a	n/a	
**Albumin**	0.92	0.9–0.95	< 0.001	0.96	0.93–1.0	0.0299
**CRP**	1.00	1.0–1.01	< 0.001	1.00	1.00–1.00	0.0208

*CKD* Chronic Kidney Disease, *CVD* cardiovascular disease, *ACE-I* angiotensin-converting enzyme inhibitor, *ARB* angiotensin II receptor blocker, *CI* confidence interval, *OR* odds ratio

^a^Variables were entered into the model when the a level of risk factor was less than 0.1

CKD was defined as baseline eGFR< 60 ml/min/1.73m^2^

### Cox regression analysis

**Fig. 2:**
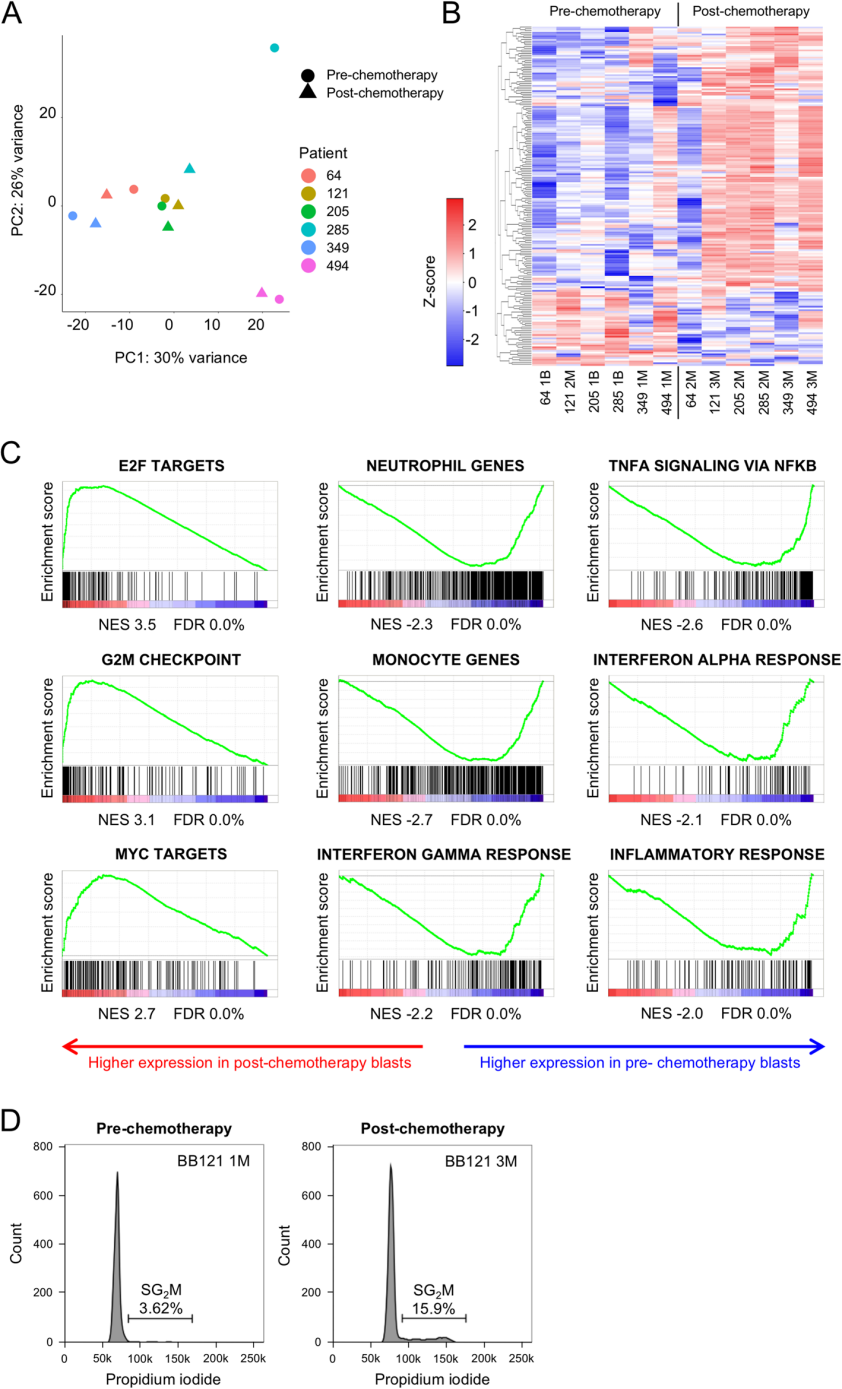
Kaplan–Meier survival curves for patients with and without AKI. Survival probability is reduced in the presence of AKI

A total of 27.9% of the whole study population died in hospital. Mortality rates were significantly higher among patients who developed AKI than the ones who did not (44.4% vs 17.3%, *p* < 0.001) (Table 1 and suppl. Table 2a). The multiple Cox regression analysis for 30-day mortality demonstrated that AKI of all stages was independently associated with risk of death at 30 days (OR 1,59; 95%CI 1.19–2.13, *p* = 0.016 for AKI stage 1, OR 2.71; 95%CI 1.82–4.05, p < 0.001 for AKI stage 2, OR 2.99; 95%CI 2.17–4.11, p < 0.001 for AKI stage 3) (Table 4a). The increased mortality associated with AKI is also illustrated in the Kaplan-Meier curve (Fig. 2). In order to account for potential bias from selection of admission for patients with more than one admission during that period of time, we performed a sensitivity analysis on mortality excluding them (*n* = 17), which suggested similar results (Table 4b and suppl. Table 2b).

**Table 4:** Cox Regression Analyses

Variable	Unadjusted HR	95% CI	p value	Adjusted HR^b^	95% CI	p value
a. Univariate and multivariate Cox regression analyses of AKI associated with mortality at 30 days						
**AKI (all stages)**	2.67	2.14–3.34	0.0000		n/a	
**No AKI**	Ref	Ref	Ref	Ref	Ref	Ref
**AKI stage 1**	2.24	1.71–2.94	0.0000	1.59	1.19–2.13	0.0018
**AKI stage 2**	4.36	3.00–6.34	0.0000	2.71	1.82–4.05	0.0000
**AKI stage 3**	2.79	2.11–3.70	0.0000	2.99	2.17–4.11	0.0000
b. Univariate and multivariate Cox regression analyses of AKI associated with mortality at 30 days - sensitivity analysis						
**AKI (all stages)**	2.67	2.14–3.34	0.0000		n/a	
**No AKI**	Ref	Ref	Ref	Ref	Ref	Ref
**AKI stage 1**	2.26	1.72–2.97	0.0000	1.58	1.18–2.12	0.0020
**AKI stage 2**	4.25	2.91–6.2	0.0000	2.61	1.74–3.92	0.0000
**AKI stage 3**	2.77	2.09–3.67	0.0000	2.95	2.14–4.06	0.0000

*AKI* acute kidney injury, *HR* hazard ratio, CI, confidence interval

^a^Variables were entered into the model when the level of risk factor was less than 0.1

^b^adjusted for age, gender, comorbidities (chronic kidney disease, hypertension, cardiovascular disease, diabetes, malignancy, neurological disease, lung disease) and inflammatory markers (CRP, albumin, Neutrophil: Lymphocyte ratio)

## Discussion

In this cohort study conducted across two sites of a large tertiary centre in London, UK, we demonstrated a high AKI incidence of 39% in patients hospitalised for COVID-19. Acute kidney injury appeared to be an independent risk factor for mortality across all stages of severity, including stage 1.

To the best of our knowledge this is the largest patient series from the UK reporting on COVID-19 AKI, and is the only study to report historical baseline renal function and long-term renal outcomes. Hamilton et al, who also reported data from the UK, demonstrated a lower AKI rate of 20.3% among patients hospitalised with COVID-19, however there were no data available on historical renal function and admission creatinine was considered as baseline, which might have led to underreporting of AKI incidence [7]. In our study one of the main determinants of increased AKI risk was pre-existing CKD.

### AKI prevalence and incidence rates

Our findings of high AKI rates in patients with COVID-19 are consistent with large US patient series (32–46%) [5, 6, 16]. Combined data from 13 New York (NY) hospitals (total *n* = 9657), demonstrated that AKI occurred in 39.9%, with stage 1 in 17% of the whole cohort, stage 2 in 8.7% and stage 3 in 14.2% [5, 6, 16, 17]. Nevertheless, some early reports, mainly from China, suggested considerably lower rates (0–7%) [1–4, 18, 19]. A meta-analysis of 142 studies (total *n* = 49,048) reported a pooled AKI incidence of 28.6% (95%CI 19.8–39.5) among 20 studies based in the USA and Europe, and a much lower 5.5% (95%CI 4.1–7.4) among 62 studies from China [20]. Several factors may account for the variation in reported AKI rates. In most studies demonstrating low AKI rates, the reported baseline CKD rates were lower (0.7–4.3%) [2–4, 18, 19] in comparison to ours and the large US patient series [5, 6, 16]. It is difficult to determine, if these differences are related to variation in definitions and methods of recording CKD or patient characteristics among geographic areas. Furthermore, it has been hypothesised, that this might be due to considerable variation in the admission criteria between China and Europe and US, and, therefore, differences in COVID-19 severity among study populations [20]. Lastly, there has been substantial inconsistency among definitions of baseline renal function used in previous studies [2, 3, 18, 21].

It was noted that the weekly AKI incidence rate increased over time to peak towards the middle of the study period and then declined reaching its nadir at the end of the observation period. This observation may be relevant to findings of Navaratnam et al, who suggested temporal changes of COVID-19 in-hospital mortality in England [22] with reducing rates towards May 2020. Several factors such as demographic, socioeconomic and clinical practice-related may have contributed and warrant further investigation on a broader scale.

### ICU patient subgroup

The majority of patients admitted to ICU from our cohort sustained a stage 3 AKI (67%), while more than half of all ICU patients (54%) required RRT at some point. Some US patient series have reported AKI rates in ICU of more than 60% (61–78%) with RRT requirements for 35 to 50.9% of all patients [23–25]; however, others reported lower rates (4 to 23%) [6, 26, 27]. In our centre, an increased use of PD in ICU occurred during the study period due to a shortage of CVVHDF capacity [28].

It is possible that high AKI rates in this patient cohort are partly driven by a hyperinflammatory response, as suggested in a recent analysis indicating distinct biological response patterns in COVID-19, with renal injury linked to an enhanced inflammatory state [29].

Notably, the RRT rate in our ICU cohort is considerably higher compared to the contemporaneous (up to June 2020) rate reported by the Intensive Care National Audit and Research Centre (ICNARC) in the UK according to which, 26.2% among 9132 patients with COVID-19 required RRT at some point (5.1% of which had pre-existing ESRD on dialysis) [30]. It is not possible to ascertain what this discrepancy on RRT rates may be attributed to; however, it is worth noting that there was a considerably higher representation of black ethnicity in our ICU cohort of 30.5% as compared to 9.2% in ICNARC data.

### AKI risk factors

The strongest independent determinant of AKI was the presence of pre-admission CKD defined as eGFR< 60 ml/min/1.73m^2^, which increased the risk of developing AKI more than three-fold. An association with pre-admission CKD has been confirmed in other reports, including a large meta-analysis of 142 studies [5, 16, 20]. Chan et al. have similarly reported a 3-fold increase of risk for AKI in the presence of pre-admission eGFR< 60 ml/min/1.73m^2^among patients hospitalised with COVID-19 (*n* = 3993) [5], while Bowe et al suggested a step-wise increase in AKI risk with CKD stage in their retrospectively studied US Veteran cohort (*n* = 5216) [16]. In addition to cardiac failure, pre-existing CKD was the only independent predictor for AKI reported by Kohle et al [31].

In our study, inpatient diuretic use was associated with a 79% higher AKI risk independent of demographics and comorbidities. Given the persisting uncertainties with regards to the management of COVID-19 AKI, caution in relation to the use of diuretics, especially in the presence of other AKI risk factors and background renal impairment may be prudent. However, due the observational nature of this study, we can only infer an association, and not a definitive causal link, between diuretic usage and AKI, and management decisions with regards to diuretic use should be made on an individual patient basis.

The changing AKI incidence rates in association with the link between inpatient diuretic use and AKI risk, may imply that a change in clinical practice and management of COVID-19 cases (in terms of fluid balance and diuretic prescription) may have partly played a role.

It is possible that early on during the pandemic, there was concern that COVID-19 patients were at risk of capillary leak linked to the hyperinflammatory state and consequently clinicians were cautious about excess fluid replacement. As the pandemic unfolded, the clinical community became rapidly aware of the high rates of AKI and clinical picture of volume depletion that evolved during the course of the disease. In the absence of published data, our local practice changed as we developed more experience in managing these patients during the first wave, adopting a more liberal fluid management strategy and actively withholding diuretics upon admission.

Black ethnicity and hypertension were also independent predictors of AKI in our study, which is consistent with other reports [6, 16]. In a previous meta-analysis on AKI (non-COVID), black ethnicity was predictive of increased AKI rates at higher eGFR levels [32] while it has been suggested that the higher AKI (non-COVID) risk associated with black ethnicity, becomes less pronounced when adjusting for socioeconomic disparities [33]. Interestingly, there was no link between high CKD-risk apolipopotein1 (APOL1) variants and AKI [33].

### Impact of AKI on mortality and renal outcomes

In our study, the occurrence of AKI was independently associated with increased mortality at 30 days post-admission with a notably higher risk with increasing severity, with patients sustaining a stage 3 AKI carrying a 3-fold higher risk of death compared to their counterparts without AKI. The association of AKI with poor prognosis is well-described [34] and has been confirmed in COVID-19 [5, 16, 17, 19, 35]. The majority (84.0%) of patients with AKI in our cohort that were discharged alive recovered renal function to their pre-admission levels, while the respective percentage was lower (69.7%) for the most severe stage 3. This high recovery rate is in contrast to those reported by other studies (renal recovery to pre-admission levels 53–65% [5, 16]. None of our patients required RRT following discharge, while other studies have reported ongoing RRT requirements of 20% [16] and 30.6% [17] of the subgroup that required RRT during admission. With regards to longer term outcome, we present 3-to-6-month follow-up up data for the majority of survivors with AKI3. Among those, 30.7% presented a persistent decline in eGFR of > 15 ml/min/1.73m^2^ at 3 to 6 months. Moreover, 21.5% without pre-existing renal impairment developed an eGFR of 60 < ml/min/1.73m^2^. This suggests that the burden of CKD following COVID-19-related AKI may be substantial and under-diagnosed in the population as a whole, with significant future implications for both renal service provision and the associated higher risk of cardiovascular disease [36].

## Conclusions

In our study, we have shown that among patients hospitalised for COVID-19 in a large tertiary centre of London in the first half of 2020, a significant proportion developed AKI with considerable implications. The changing incidence pattern over time may reflect temporal changes in severity of case-mix or other contributing factors. The main predisposing factor for the development of AKI was pre-existing renal impairment, highlighting the importance of increased awareness among healthcare professionals and patients. Though, AKI appears to be part of a more severe COVID-19 presentation, certain treatment approaches, such as the use of diuretics, possibly aggravate this, and therefore caution may be warranted especially in higher risk patients with underlying CKD. Despite encouraging short-term outcomes with regards to renal recovery, one third of survivors with AKI3 presented newly established renal impairment at 3 to 6 months and the overall long-term burden with regards to CKD and cardiovascular disease remains to be seen.

### Limitations and future perspectives

This study is observational and therefore cannot infer causality. Mainly due to increased mortality rate in the patient subgroup with AKI stage 3 follow up creatinine data were available for 65 patients. A larger follow-up programme would be needed to definitively determine the long-term impact of this disease. The changing AKI incidence patterns could prompt investigation of contributing variables, further to established AKI risk factors, to inform clinical practice during next stages of the pandemic.

## Supplementary Information

**Additional file 1.** (PPTX 62 kb)

**Additional file 2.** Suppl. Table 1. Further laboratory results and clinical characteristics. Suppl. Table 2. Univariate and multivariate Cox regression analyses of risk factors associated with mortality at 30 days. Suppl. Table 2b. Univariate and multivariate Cox regression analyses of risk factors associated with mortality at 30 days - sensitivity analysis.

## Data Availability

The datasets used and/or analysed during the current study are available from the corresponding author on reasonable request.
