# Supplementary material for: COVID-19-related acute kidney injury; incidence, risk factors and outcomes in a large UK cohort
Source: BMC Nephrol. 2021 Nov 1;22:359. doi: 10.1186/s12882-021-02557-x (PMC8557997; doi:10.1186/s12882-021-02557-x)
Supplement: Supplementary file 1 — Additional file 1. (PPTX 62 kb) [file 12882_2021_2557_MOESM1_ESM.pptx]

## Slide 1
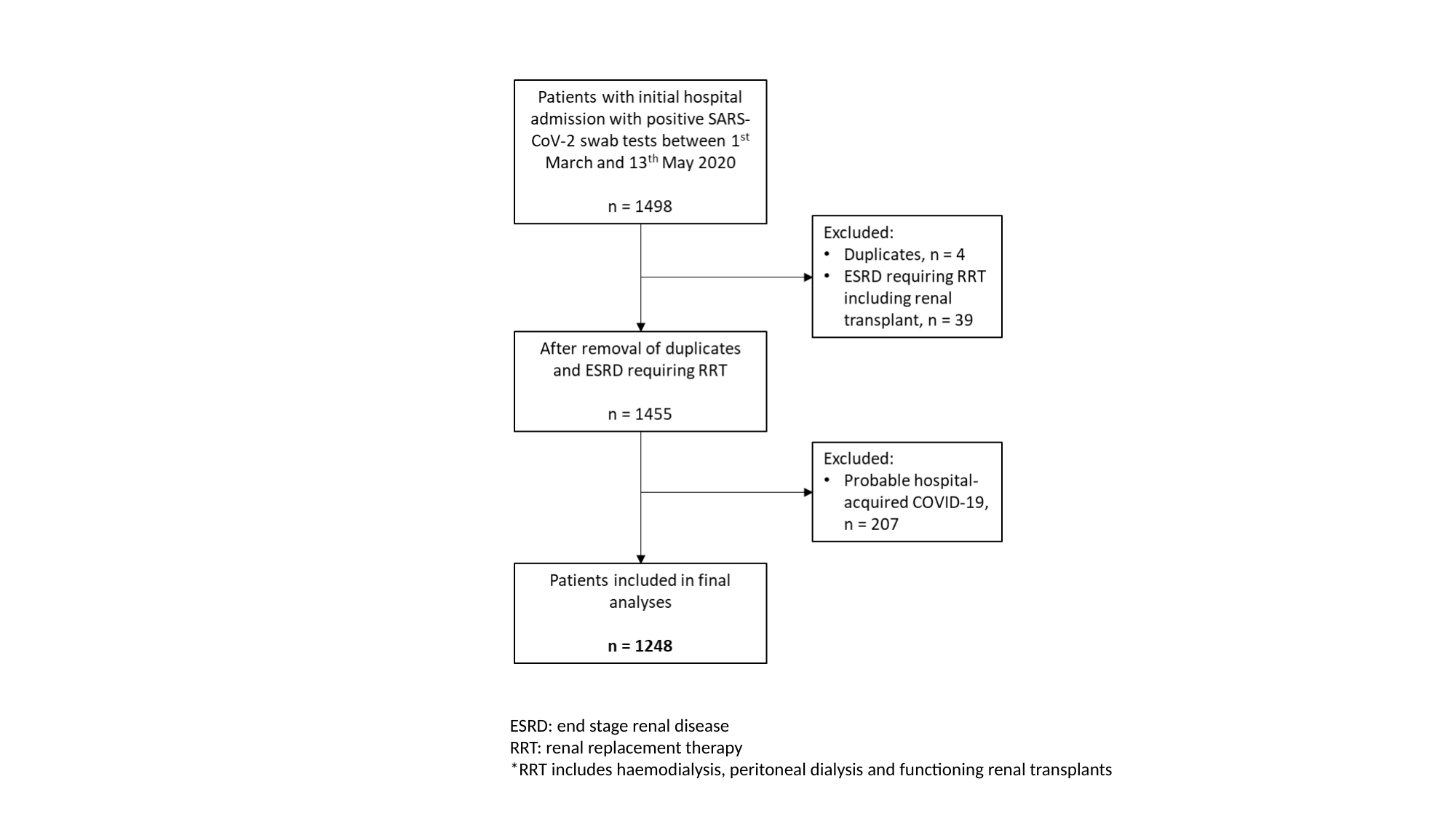

ESRD: end stage renal disease
RRT: renal replacement therapy
*RRT includes haemodialysis, peritoneal dialysis and functioning renal transplants
