# Supplementary material for: COVID-19-related acute kidney injury; incidence, risk factors and outcomes in a large UK cohort
Source: BMC Nephrol. 2021 Nov 1;22:359. doi: 10.1186/s12882-021-02557-x (PMC8557997; doi:10.1186/s12882-021-02557-x)
Supplement: Supplementary file 2 — Additional file 2. Suppl. Table 1. Further laboratory results and clinical characteristics. Suppl. Table 2. Univariate and multivariate Cox regression analyses of risk factors associated with mortality at 30 days. Suppl. Table 2b. Univariate and multivariate Cox regression analyses of risk factors associated with mortality at 30 days - sensitivity analysis. [file 12882_2021_2557_MOESM2_ESM.docx]

**Supplemental Material**

**Suppl. Table 1. Further laboratory results and clinical characteristics**

|  | **All (n=1248)** | | **No AKI (n=761)** | | **All AKI (n=487)** | | **AKI1 (n=248)** | | **AKI2 (n=64)** | | **AKI3 (n=175)** | | **p^*^** | | **p**^⁋^ **for trend** | |  |
| --- | --- | --- | --- | --- | --- | --- | --- | --- | --- | --- | --- | --- | --- | --- | --- | --- | --- |
| **Laboratory results** | |  | |  | |  | |  | |  | |  | |  | |  | |
| **Urea**(mmol/l),Median (IQR) | 7.1 (4.6, 11.8) | | 5.6 (4.0, 8.1) | | 11.8 (7.2, 19.5) | | 11.6 (7.1, 16.7) | | 16.1 (9.8, 24.1) | | 10.5 (6.0, 21.2) | | < 0.001 | | < 0.001 | |  |
| **Sodium**(mmol/l), Median (IQR) | 137 (134, 140) | | 137 (134, 140) | | 137.0 (134.0, 141.0) | | 137.0 (134.0, 141.0) | | 138.0 (134.8, 146.2) | | 137.0 (133.0, 141.0) | | 0.09 | | 0.117 | |  |
| **Peak D-dimer**(mg/l),Median (IQR) (n=529) | 1553 (800, 3821) | | 1219 (670, 2222) | | 3310 (1330, 6660) | | 1865 (831, 3513) | | 2006 (1009, 4270) | | 5780 (3360, 7939) | | < 0.001 | | < 0.001 | |  |
| **Peak troponin**(ng/l),Median (IQR) (n=520) | 30.5 (13.0, 80.0) | | 19.0 (8.0, 39.0) | | 59.0 (28.2, 132.0) | | 38.0 (20.0, 94.5) | | 47.0 (31.0, 86.0) | | 86.0 (36.0, 220.0) | | < 0.001 | | < 0.001 | |  |
| **Peak CK, (UI/l)** Median (IQR) (n=350) | 228.5 (85.0, 649.2) | | 128.0 (68.0, 312.5) | | 381.0 (164.2, 1405.8) | | 262.0 (121.5, 535.5) | | 240.0 (111.0, 774.5) | | 626.0 (231.2, 2123.0) | | < 0.001 | | < 0.001 | |  |
| **Abnormal chest radiograph,** n (%) | 1077 (86.8%) | | 640 (84.8%) | | 437 (89.9%) | | 219 (88.7%) | | 56 (87.5%) | | 162 (92.6%) | | 0.029 | | 0.157 | |  |
| **ICU cohort,** n (%) (data available for n=131) |  | |  | |  | |  | |  | |  | |  | |  | |  |
| Mechanical ventilation | 127 (96.9%) | | 23 (92.0%) | | 104 (98.1%) | | 4 (100.0%) | | 3 (75.0%) | | 97 (99.0%) | | 0.11 | | 0.018 | |  |
| Vasopressors or inotropes | 82 (88.2%) | | 16 (76.2%) | | 66 (91.7%) | | 1 (33.3%) | | 3 (100.0%) | | 62 (93.9%) | | 0.053 | | 0.003 | |  |
| ECMO | 10 (7.6%) | | 0 (0.0%) | | 10 (9.4%) | | 1 (25.0%) | | 0 (0.0%) | | 9 (9.2%) | | 0.11 | | 0.218 | |  |

AKI, acute kidney injury;IQR, interquartile range; CK, creatine kinase; PE, pulmonary embolism; DVT, deep vein thrombosis; ICU, intensive care unit; ECMO, Extracorporeal membrane oxygenation

**^*^**comparison between AKI vs non-AKI

^⁋^comparison across AKI stage subgroups

**Suppl. Table 2. Univariate and multivariate Cox regression analyses of risk factors associated with mortality at 30 days**

| **Variable** | **Unadjusted HR** | **95% CI** | **p value** | **Adjusted HR** | **95% CI** | **p value** |
| --- | --- | --- | --- | --- | --- | --- |
| **Age** | 1.04 | 1.04-1.05 | 0.0000 | 1.04 | 1.03-1.05 | 0.0000 |
| **Male sex** | 1.36 | 1.09-1.71 | 0.0076 | 1.40 | \| 1.10-1.78 \| 1.80 \| \| --- \| --- \| | 0.0055 |
| **Race: White ethnicity** | Ref | Ref | Ref | Ref | Ref | Ref |
| **Black** | 0.66 | 0.50-0.86 | 0.0023 | 0.87 | 0.65-1.18 | 0.3852 |
| **Asian** | 0.72 | 0.47-1.09 | 0.1232 | 0.95 | 0.62-1.47 | 0.8326 |
| **Mixed/Other** | 0.67 | 0.41-1.08 | 0.1020 | 1.11 | 0.66-1.87 | 0.6914 |
| **Unknown** | 0.56 | 0.36-0.88 | 0.0122 | 0.85 | 0.53-1.36 | 0.5016 |
| **CKD** | 2.32 | 1.86-2.9 | 0.0000 | 1.40 | 1.09-1.79 | 0.0090 |
| **Hypertension** | 1.49 | 1.19-1.86 | 0.0005 | 0.84 | 0.66-1.08 | 0.1764 |
| **CVD** | 1.77 | 1.42-2.20 | 0.0000 | 1.00 | 0.79-1.28 | 0.9715 |
| **Diabetes** | 1.07 | 0.85-1.34 | 0.5870 |  |  |  |
| **Malignancy** | 1.72 | 1.33-2.22 | 0.0000 | 1.26 | 0.96-1.66 | 0.1022 |
| **Neurological disease** | 2.09 | 1.71-2.94 | 0.0000 | 1.66 | 1.30-2.12 | 0.0000 |
| **Lung disease** | 1.21 | 0.96-1.54 | 0.1130 | 1.32 | 1.03-1.70 | 0.0286 |
| **Albumin** | 0.90 | 0.89-0.92 | 0.0000 | 0.94 | 0.92-0.97 | 0.0000 |
| **CRP** | 1.00 | 1.00-1.00 | 0.0000 | 1.00 | 1.00-1.0 | 0.0004 |
| **Neutrophil: Lymphocyte ratio** | 1.03 | 1.02-1.04 | 0.0000 | 1.00 | 0.99-1.01 | 0.7664 |

CKD, Chronic Kidney Disease; CVD, cardiovascular disease; ACE-I, angiotensin-converting enzyme inhibitor; ARB, angiotensin II receptor blocker; CI, confidence interval; HR, hazard ratio.

CKD was defined as baseline eGFR< 60ml/min/1.73m^2^

^a^Variables were entered into the model when the a level of risk factor was less than 0.1.

**Suppl. Table 2b. Univariate and multivariate Cox regression analyses of risk factors associated with mortality at 30 days - sensitivity analysis.**

| **Variable** | **Unadjusted HR** | **95% CI** | **p value** | **Adjusted HR** | **95% CI** | **p value** |
| --- | --- | --- | --- | --- | --- | --- |
| **Age** | 1.04 | 1.04-1.05 | 0.0000 | 1.04 | 1.03-1.05 | 0.0000 |
| **Male sex** | 1.35 | 1.07-1.69 | 0.0010 | 1.38 | \| 1.09-1.76 \| 1.80 \| \| --- \| --- \| | 0.0008 |
| **Race: White ethnicity** | Ref | Ref | Ref | Ref | Ref | Ref |
| **Black** | 0.66 | 0.50-0.86 | 0.002 | 0.88 | 0.65-1.19 | 0.395 |
| **Asian** | 0.71 | 0.46-1.09 | 0.115 | 0.96 | 0.62-1.48 | 0.839 |
| **Mixed/Other** | 0.67 | 0.41-1.09 | 0.108 | 1.11 | 0.66-1.87 | 0.685 |
| **Unknown** | 0.56 | 0.36-0.88 | 0.013 | 0.86 | 0.54-1.36 | 0.532 |
| **CKD** | 2.36 | 1.89-2.95 | 0.0000 | 1.40 | 1.09-1.79 | 0.0090 |
| **Hypertension** | 1.51 | 1.21-1.89 | 0.0000 | 0.86 | 0.67-1.10 | 0.227 |
| **CVD** | 1.78 | 1.43-2.21 | 0.0000 | 1.00 | 0.79-1.28 | 0.980 |
| **Diabetes** | 1.07 | 0.85-1.34 | 0.5700 |  |  |  |
| **Malignancy** | 1.74 | 1.35-2.25 | 0.0000 | 1.26 | 0.95-1.65 | 0.104 |
| **Neurological disease** | 2.09 | 1.68-2.60 | 0.0000 | 1.65 | 1.29-2.11 | 0.0000 |
| **Lung disease** | 1.22 | 0.96-1.55 | 0.102 | 1.33 | 1.04-1.71 | 0.025 |
| **Albumin** | 0.90 | 0.89-0.92 | 0.0000 | 0.95 | 0.92-0.97 | 0.0000 |
| **CRP** | 1.00 | 1.00-1.00 | 0.0000 | 1.00 | 1.00-1.0 | 0.0000 |
| **Neutrophil: Lymphocyte ratio** | 1.03 | 1.02-1.04 | 0.0000 | 1.00 | 0.99-1.01 | 0.800 |

CKD, Chronic Kidney Disease; CVD, cardiovascular disease; ACE-I, angiotensin-converting enzyme inhibitor; ARB, angiotensin II receptor blocker; CI, confidence interval; HR, hazard ratio.

CKD was defined as baseline eGFR< 60ml/min/1.73m^2^

^a^Variables were entered into the model when the a level of risk factor was less than 0.1.
